# Medicine and Phlebolymphology: Time to Change?

**DOI:** 10.3390/jcm9124091

**Published:** 2020-12-18

**Authors:** Attilio Cavezzi

**Affiliations:** Eurocenter Venalinfa, 63074 San Benedetto del Tronto, Italy; info@cavezzi.it

**Keywords:** chronic venous diseases, varicose veins, vein thrombosis, venous ulcer, lymphedema, endovenous treatments, sclerotherapy, surgery, compression therapy, cost-effectiveness, integrative medicine

## Abstract

Biomedical science is undergoing a reappraisal of its scientific advancement process and of the related healthcare management. Progress in medicine should combine improvements of knowledge, efficacy, and safety of diagnostic/therapeutic procedures, with adequate cost-effectiveness profiles. This narrative review is aimed at assessing in medicine, more specifically in phlebology and lymphology: (a) scientific literature possible biases, (b) the level of evidence, comprehensiveness, and cost-effectiveness of the main therapeutic options, and (c) the possible contribution of integrative and translational medicine. Current medical research may have cognitive biases, or industry-tied influences, which impacts clinical practice. Some reductionism, with an increasing use of drugs and technology, often neglecting the understanding and care of the root causative pathways of the diseases, is affecting biomedical science as well. Aging brings a relevant burden of chronic degenerative diseases and disabilities, with relevant socio-economic repercussions; thus, a major attention to cost-effectiveness and appropriateness of healthcare is warranted. In this scenario, costly and innovative but relatively validated therapies may tend to be adopted in venous and lymphatic diseases, such as varicose veins, leg venous ulcer, post-thrombotic syndrome, pelvic congestion syndrome, and lymphedema. Conversely, a more comprehensive approach to the basic pathophysiology of chronic venous and lymphatic insufficiency and the inclusion of pharmacoeconomics analyses would benefit overall patients’ management. Erroneous lifestyle and nutrition, together with chronic stress-induced syndromes, significantly influence chronic degenerative phlebo-lymphatic diseases. The main active epigenetic socio-biologic factors are obesity, dysfunctions of musculo-respiratory-vascular pumps, pro-inflammatory nutrition, hyperactivation of stress axis, and sedentarism. An overall critical view of the scientific evidence and innovations in phebolymphology could be of help to improve efficacy, safety, and sustainability of current practice. Translational and integrative medicine may contribute to a patient-centered approach. Conversely, reductionism, eminence/reimbursement-based decisional processes, patients’ lack of education, industry-influenced science, and physician’s improvable awareness, may compromise efficacy, safety, appropriateness, and cost-effectiveness of future diagnostic and therapeutic patterns of phlebology and lymphology.

## 1. Scientific Progress and Chronic Degenerative Diseases

Biomedical science deals with the sum of all the psycho-biological changes that affect the individual in the course of life. Cell senescence features the lifelong negative effects on human beings’ efficiency, functional reserves, and psycho-physical homeodynamic adaptation to inner and outer/environmental changes [1].

Evidence from epigenetics shows the strong correlation between social and physical environment on overall human health and disease [2]. This proven influence of nutrition, lifestyle, life events, and environment on gene expression, remarkably contributes to chronic degenerative diseases (CDD). A more integrative and comprehensive view is slowly paving the way to a functional biomedical approach, to deal with the root processes beyond the symptoms and signs of a determined disease, thus moving away from a possible reductionist medicine. An editorial of “The Lancet” defined human health as “the ability to adapt to inner and outer changes” [3].

Of importance, recently, the social cost of the current biomedical approach has become increasingly less sustainable. An exponential increase has been documented in the prevalence of several CDD, such as cancer, neurodegeneration, cardiovascular and cerebral atherosclerosis, obesity, diabetes, and autoimmune diseases, in the general population and especially in the elderly. Consequently, due to this cumulative and overwhelming pathologic burden which results in a dramatic increase in patient disability and generalized consumption of health resources, healthcare systems are suffering from sustainability issues.

Venous and lymphatic diseases also show a growing prevalence in the population, with obvious global socio-economic repercussions on healthcare management. Phlebology and lymphology are certainly not immune to the risk of lack of cost-effectiveness, reductionism, and the biases that affect biomedical science in other areas of research. In fact, in the last decade, the literature reviews have proposed some re-appraisal of guidelines, recommendations, and good clinical practice regarding the care of acute and chronic venous disease (CVD) and of lymphedema (LYM).

Progress in medicine strictly relates to the scientific evidence derived from literature data; similarly, clinical practice reflects the continuous improvement of scientific knowledge. However, several interplaying socio-environmental-economic factors significantly influence all these aspects of health management.

This narrative review intends to provide a series of data and speculations concerning some of the scientific and socio-economic issues related to the advancements in biomedical science and, more in depth, in venous and lymphatic diseases.

### 1.1. Methodology

Following the process of critical analysis of medical literature, a critical review of the publications inherent to phlebolymphology has been performed in this review.

A literature search was performed to retrieve pertinent articles in PubMed, Google Scholar, Research Gate, and Cochrane library from the last 20 years. The documents were selected from systematic reviews, reviews, meta-analyses, consensus documents and guidelines, and randomized controlled trials. Appendix A summarizes the main headings and keywords that were utilized to find the relevant documents in the existing literature.

### 1.2. Integrative Medicine, Aging, and Healthcare Costs

The concepts of a more comprehensive diagnostic and therapeutic pathway and cost-effective choices in healthcare, are gradually permeating medical research and practice.

Nowadays, overweight and obesity affect 2 to 2.5 billion people worldwide; just in the USA, it is estimated that by 2030, half the population could suffer from this disease, which would result in a 549.5 billion USD increase of expenditure in comparison to 2010 obesity-related costs [4].

These data have a repercussion on a series of associated or consequent non-transmissible diseases, also determining a much higher vulnerability to other transmissible ones (e.g., Covid-19) [5]. Neglecting this extremely important health question may lead to several complex biomedical and socio-economic issues in the early future, which will invariably pertain to vascular disciplines as well.

Notwithstanding the relevant pharmacological/technological advancements, the maximum duration of human life has not increased, whereas the average lifespan significantly increased in the last century. The extension of the average life, together with the non-corresponding improvement of health-span in the elderly, are objectively causing exponentially increasing problems to our civilization.

Human diseases seem to depend upon the chromosomal heritage for about 25% [6], and the residual 75% of pathological processes basically depends on epigenetics; thus, the individual’s everyday life experiences and choices, regarding nutrition, lifestyle, and psychosocial conditions, condition cell metabolic pathways through epigenetic mechanisms. This definitely proven role of the uncountable epigenetic factors, which have a lifelong influence on gene expression and phenotype, regards phlebolymphopathic patients as well.

The so-called “inflammaging” (aging and cell senescence caused by chronic low-grade cellular inflammation (CLGCI) [1,7,8]), is regarded as the core process of the molecular events at the root of human aging and of CDD. Basically, phlebolymphology includes a wide spectrum of CDD, such as chronic venous insufficiency (CVI) and LYM, where CLGCI plays a determinant role [1,9].

Of interest, senescence-associated secretory phenotype (the proteins which are increasingly secreted during cell senescence) remarkably influences coagulation mechanisms [10]; hence, aging significantly raises venous thromboembolism (VTE) risk through hemostasis derangements. Similarly, lymph vessels are subject to remarkable detrimental changes during aging, which influence lymphatic flow, microvascular/tissue homeodynamics, and ultimately pathogen clearance [11].

A few main pathologic biochemical processes have proven to be at the root of cellular senescence and CLGCI (see Table 1).

López-Otín summarized both senescence mechanisms and the possible regenerative, reparative, and virtuous biochemical processes aimed at achieving a better cell resilience, the latter being considered the cornerstone for human health [12].

Besides conventional pharmacological and technological support, many validated interventions have been recognized as fundamental pillars in a framework of an integrative treatment of CLGCI and CDD. Most of these interventions are based on lifestyle, nutrition, and stress management [1,12].

Table 2 summarizes the most referenced integrative medicine interventions (beyond drugs and technologies), which may be of help in CDD, hence in several phlebolymphology syndromes.

Regrettably, daily medical practice often shows a reductionist strategy, which is based on the diagnostic/therapeutic approach to a patient’s sole symptoms and signs, thus neglecting basic deranged pathophysiologic mechanisms.

In view of the concept of science as “a constantly changing basis of knowledge” [13], scientific research should take into account this elusive and limited nature of scientific progress; consequently, a more balanced view of any innovative diagnostic/therapeutic step would be desirable within the medical community.

Bird suggests that scientific progress may relate to an augmentation of scientific knowledge for a few scientists, but also to an increase of verisimilitude, understanding, and of problem-solving for others [14]. More specifically, it is somehow agreed that progress in medicine occurs when it brings an improvement of efficacy and safety of diagnostic/therapeutic procedures, in combination with an adequate (possibly better) cost-effectiveness profile.

Pharmacoeconomics address relevant issues in healthcare management, assessing effectiveness and costs of medical interventions, principally drugs [15]. In the last 20 years, the number of PubMed-included pharmacoeconomics yearly citations grew from 641 to 1880. This type of analysis investigates medical diagnostic/therapeutic innovations in terms of cost-benefit, cost-effectiveness, cost-minimization, cost-of-illness, and cost-utility. Basically, any new treatment could be defined more or less effective/expensive in comparison to the available and comparable validated interventions. Beyond the obvious positive impact of pharmacoeconomics on the scientific community, several institutions may benefit from this cultural approach to design proper strategies in healthcare provision [16]. Covid-19 is imposing socio-economic restrictions worldwide, thus providing a further motivation to reflect upon the need to improve the cost/benefit rationale of most medical care strategies.

### 1.3. Biases in Scientific Research and Clinical Practice

Evidence-based medicine (EBM) is the basis for the best medical practice today. Evidence, in the form of acquisitions from newly observed phenomena, is the instrument by which science supersedes old theories with innovative knowledge. This process of constant revision of scientific knowledge inevitably determines moments of transition in which diagnostic and therapeutic proposals, or explanations of pathophysiology, may coexist, without having a full agreement among scientists. Phlebology and lymphology are nowadays in a phase where the decisional process is affected by some uncertainties in a few scopes, and in which they suffer from the application of suboptimal knowledge. In this view, a more careful assessment of expensive innovations seems to be reasonable and also necessary due to the interplay of biases and other factors that we proceed to discuss. In 2005, Ioannidis published the currently most-accessed article in the history of PLOS (public library of science) [17], titled “Why Most Published Research Findings are False” [18], where he listed a series of biases and controversies which may compromise scientific research and publications. Scientific progress seemingly suffers from a series of inherent biases, cognitive or otherwise, which may bring scientists and researchers to take mental shortcuts and/or may inhibit their necessary critical sense, thus introducing the risk of misleading research and induce to improper clinical decisions. Other scientists pointed out the necessity to agree on the assessment of biases within clinical research [19], so as to control human interference on data collection and elaboration; more specifically, the aim would be to reduce methodological flaws, limitations, and inadequate practices in scientific research.

The most common cognitive biases in scientific research [20] are listed in Table 3.

Bias may also manifest due to industry aid. Economic and logistic support from industries is correctly considered an instrumental and fundamental help, to finance otherwise extremely expensive and complex research. Hence, in the last decades, we had a proliferation of industry-backed scientific studies, which highlighted, with some evidence, new and “better” diagnostic and therapeutic proposals in biomedical fields.

Of interest, a limited value of some of these industry-sponsored proposals has been highlighted, especially in terms of cost-benefit improvement [21]. Furthermore, several scientists have raised both the issue of unpublished industry-funded studies due to their negative results [22], and the question of retracted studies after their publication, due to scientific misconduct (72%) and errors (28%) [23]. Finally, a discrepancy between the outcomes of independent studies and the ones published in industry-sponsored studies may exist [24].

Goldacre, in his book, suggests the existence of an intruding effect of corporate marketing strategies into clinical practice [25]. If, on one side, biomedical company profit is fundamental to sustain scientific research, on the other side, it would be desirable to avoid that the logic of profit may restrict the freedom and the objectiveness of research.

Lastly, a plea to combat corruption in global healthcare was recently made in a few eminent publications [26,27,28], which witnesses the need for a stronger attention over a few specific aspects of medical progress, as reported above.

### 1.4. Medical Education Issue

Nowadays, medical education relies on a relevant acquisition of pharmaceutical knowledge, together with some acquaintance with the useful technologies. Basic pathophysiology of most diseases are barely represented within the current curricula in medical training, whereas a systematic treatment of symptoms and signs of the diseases is advocated. Similarly, the detrimental role of erroneous nutrition, lifestyle, and chronic stress is under-emphasized in current biomedical education.

The suboptimal public financing allocation for independent medical research is another issue to be considered as well. A tendency towards the overestimation of the biochemistry/genetic aspect of medical diseases may compromise the necessary focus on the clinical approach to our patients. As a result, physicians may have a limited opportunity to achieve a deeper knowledge of relevant information related to epidemiology, efficacy, and safety of clinical practice.

## 2. “Modern” Phlebology and Lymphology Issues

The prevalence of chronic venous diseases (CVD) in the population of industrialized countries is estimated at around 50% [29]. The global burden of CVD is expected to dramatically increase with the higher longevity of the population and with the increase of the risk factors (e.g., obesity, disability, sedentarism) [30]. A similar scenario is extremely probable for lymphatic diseases, though sound epidemiology data on LYM are still missing. It is possible to anticipate that scientific research and care provision in phlebolymphology will be strongly related to cost-effectiveness in the future, in search of an adequate decisional process.

In the last 10 years, Cochrane reviews and a series of scientific publications concerning veins and lymphatics highlighted the absolute need for more evidence, prior to advancing further diagnostic and therapeutic recommendations.

Pharmacoeconomics studies are entering phlebology and lymphology as well, in order to measure costs, outcomes, and quality-adjusted life-years (QALY) parameters [31,32,33]. The relationship among costs, effectiveness, utility, safety, and benefit in chronic venous and lymphatic diseases is an objectively complex issue to investigate. In fact, several variables must be considered in cost-effectiveness studies, and patient’s quality of life (QOL) is becoming a core reference to assess medical care. Subjective (e.g., wellbeing) and objective (e.g., physical and mental state) parameters are being more frequently included in QOL evaluation in phlebolymphology. A few CVD-related instruments are purposely used as well, but still in a limited manner and with some debate.

Notwithstanding the current body of evidence, summarized in a few international guidelines [34,35,36], phlebology and lymphology are undergoing a constant update process of old and new proposals. Of interest, within vascular disciplines, several innovative diagnostic and therapeutic approaches happened to receive little to no attention even after a few years from their introduction and use. Furthermore, a more comprehensive approach to patients with arterial, venous, and lymphatic diseases is advocated in order to diagnose and treat many interplaying harmful lifestyle and nutrition habits, which do represent causative/worsening factors.

While the current medical approach shows an inner risk of reductionism [37], we assist with impotence to a dramatic rise in worldwide prevalence of a long series of CDD that are pushing healthcare systems towards a possible collapse, for lack of resources. Furthermore, lack of collaboration by the patients (who are typically induced to delegate their own health to drugs, technology, and to caregivers, or wish to follow fashionable novelties), lack of a new vision in health professionals, and, ultimately, lack of critical assessment of mid/long-term outcomes, may be obstacles to a comprehensive management of chronic venous and lymphatic diseases.

The development of modern civilization has definitely impacted nutrition, lifestyle, and stress tolerance, which are the cornerstones in the health-disease processes of human beings [1]. Chronic stress and the progressive decrease of environment healthy challenges due to increasing comforts and easiness of life have significantly modified human lifestyle and resilience. Similarly, human nutrition has shifted from beneficial “natural” foods to processed foods, from “good” to “bad” (e.g., trans) fats, and from a lower to a higher quote of (mostly refined) carbohydrates. Lastly, sedentarism is increasingly spreading among newer generations.

Money-driven providers (industries) of nutritional models orientate quality and quantity of daily food intake: on one side, media and industries tend to spread detrimental nutritional habits (e.g., brain-rewarding sugar addiction), consumption of drugs, and use of expensive technology. On the other side, regrettably, these stimuli meet a low level of patients’ and health professionals’ criticism.

The pandemic “diabesity”, which features diabetes type 2, overweight, obesity, and dysmetabolism, is one of the best examples of a relevant clinical issue, which dramatically influences the course of CVI and of LYM [4,38,39].

Obesity is a CDD, which negatively influences a long series of pathologies; similarly, it is a definitely proven favoring factor of VTE, doubling its risk [40], and it correlates with worse CEAP stages [4]. Obesity impairs venous and lymphatic return through a series of proven pathologic hemodynamic and lymphodynamic mechanisms; furthermore, several obesity-related deranged metabolic and inflammatory pathways contribute to the onset and worsening of CVD and LYM. It has been repeatedly emphasized that functional venolymphatic insufficiency, in the absence of any venous or lymphatic morphologic changes, may be typically generated by obesity [4,39,41,42]. Together with the explanatory mechanical mechanisms, such as increased intra-abdominal pressure and ilio-femoral reduced venous outflow, a series of metabolic derangements occur at the metabolic level with negative repercussions, both on veins and on lymphatics. More specifically, in obese patients, an activation of coagulative cascade as well as a progressive insulin increase in combination with a systemic pro-inflammatory state have been demonstrated [39,42]. Notwithstanding this ascertained role, weight control still remains a neglected issue when treating phlebopathic and lymphopathic patients.

An estimation calculated that care and treatment for CVD account for about 2% of national healthcare budgets [38], but likely higher figures are expected in the early future due to several interplaying factors such as aging, sedentarism, innovative costly procedures, increasing risk factors such as obesity, dymetabolism, and so on.

Objectively, many venous and lymphatic diseases seem to share a predominant genetic starting component (e.g., primary varicose veins and primary LYM). Regardless of this basic feature, most pathophysiology and evolutionary processes inherent to chronic venous and lymphatic diseases strictly depend upon epigenetics. A few potential factors seem to activate relevant epigenetic pathways (based on nutrition, lifestyle, psycho-social, and environmental components) and impact the progression and prognosis of most venous and lymphatic diseases. A non-comprehensive list of these detrimental factors is reported in Table 4.

Diagnostics of venous and lymphatic diseases have been characterized by the introduction of new and often expensive methodologies, which sometimes add little knowledge to “old” and inexpensive duplex ultrasound and lymphoscintigraphy accuracy. Similarly, a series of innovative proposals to treat venous diseases have been under scrutiny in the latest years [43], raising a thorough debate on their real value in terms of cost/benefit. In fact, the treatment of varices, deep vein thrombosis (DVT), CVI, leg venous ulcer (LVU), and LYM has undergone major changes in the last decades, sometimes following the promoted fashionable technologies and drugs.

Interestingly, many of these therapeutic options were abandoned in the last years, prevalently but not exclusively, due to cost-efficacy reasons.

Experts have acknowledged that guidelines and recommendations in phlebology and lymphology may need frequent actualizations [44] as per the need to review past ascertained consensus statements and to introduce new potentially influencing evidences from the succeeding trials.

### 2.1. Varicose Veins

In phlebology, varicose vein treatment represents a paradigmatic example where nearly every single year, literature relentlessly reports about new (and frequently more expensive) thermal, mechanical, and chemical techniques, which aim to replace the previously fashionable ones. Conversely, “ancient” compression therapy and hook phlebectomy still represent extremely well-reputed and science-backed treatments. The relatively young foam sclerotherapy has progressively gained more validation, also in view of the appealing results recently achieved with catheter foam sclerotherapy (CFS), in combination with tumescence and vein irrigation (96.5% reflux-free great saphenous veins at 3 years) [45]. A recent systematic review and meta-analysis detailed literature data concerning CFS, showing potential benefits from this variant of foam sclerotherapy [46]. The favorable cost/benefit ratio is the acknowledged best feature of foam sclerotherapy, a versatile and widely practiced technique [47].

Notwithstanding the suboptimal standardization of the physician-compounded sclerosant foam (SF) through the Tessari method, the extremely low cost of this extemporary foam and its overall positive short/mid-term outcomes [48] make this relatively novel option a worldwide-accepted treatment, mostly under the form of ultrasound-guided foam sclerotherapy (UGFS). In turn, the patented licensed (non-compounded) SF has been validated through a series of studies which confirmed its safety and good efficacy at short/mid-term [49].

In view of its incurring costs (significantly higher than the extemporary SF), the industrial SF has spread only within a few public/insurance health systems, where it has been recognized as a reimbursable treatment. While further literature is expected to corroborate the efficacy and safety of this specific industrialized foam, the relevant spending difference with the more common physician-compounded SF will represent an issue in future years.

In the last 30 years, the proper exploitation of color-duplex ultrasound (CDU) investigation has completely revised most pathophysiologic concepts about varicose vein disease, providing the basis for a more tailored and conservative treatment. The multiplicity of the morpho-hemodynamic findings elicited by means of CDU in varicose limbs, together with the frequent finding of a competence of the saphenous junction and of segmental saphenous refluxes [50,51], may lead to a selective use of the saphenous vein-targeting modern and costly technologies, and inexpensive and “simple” phlebectomy could suffice in specific cases. Regrettably, there is a suboptimal medical interest in basic pathophysiology of varices of the lower limbs, due to the easiness of the current therapeutic approach which is based on “cooking or sticking” the vein regardless of the morpho-functional pre-operative pattern. Lastly, when reimbursement affects the therapeutic choice, inexpensive and under-paid valuable procedures (e.g., phlebectomy, UGFS, and CFS) are neglected in favor of modern and better-reimbursed ones.

In fact, when assessing the level of evidence in the published guidelines for each of the varicose vein treatments, a significant difference emerges as to their indications and contraindications, or in peculiar situations such as small saphenous vein therapy [44].

Interestingly, multiple reviews of varicose vein treatments are highlighting the issue of long-term high recurrence rate in the treated limb (caused by technical/strategy mistakes, natural disease evolution, etc.), together with the need for re-assessment of costs and proper indications [52,53]. Furthermore, the inter-procedures difference, in terms of saphenous occlusion rate (which is in fact just one surrogate outcome) at mid-term, is well below 10%. This minimal variation questions the value and the need for innovative and more expensive treatments, to achieve results which are in the range of the ones reported with older and inexpensive procedures.

When assessing the clinical recurrence (re-occurrence of varices in the treated limb), some 30–35% recurrence rate is still contemplated at mid-term. Higher figures of new varices in the operated limbs at 5 years follow-up (42–53%) have been reported in the recent and widely referenced CLASS trial [54].

The little available literature on long-term (10 years) outcomes account for 37–56% of new varices for surgery and/or sclerotherapy [55], whereas a 66.5% clinical and/or duplex-based recurrence was documented after stripping [56]. Another study based on clinical and Doppler assessment documented 70% overall recurrence 10 years after stripping [57]. Interestingly a CHIVA (Cure conservatrice et Hemodynamique de l’Insuffisance Veineuse en Ambulatoire) vs. stripping randomized controlled trial showed a quite lower recurrence rate for both techniques (18% and 36%, respectively) at 10 years, but the smaller (<5 mm) varices were not included in the calculated figures [58]. Lastly, a prevalence of 60% reflux just at the previous crossectomy site was evidenced by means of CDU, 34 years after stripping [59].

Of interest is whichever varicose vein ablation intuitively affects the residual venous capacity and compliance in the treated limb. A reduced calf venous compliance has been proven anyway in patients with varices of the lower limbs, and a further reduction of this important pathophysiology parameter has been documented after ablation of great saphenous vein and tributaries [60]. The decrease of venous compliance may generate an increase in transmural pressure in the residual veins, which may result in varicose vein recurrence or just in loco-regional ambulatory venous hypertension and consequent clinical manifestations, from spider veins to skin changes [61]. Beyond this speculation and the chronic evolutionary nature of varicose disease, its factual tendency to recur is to be taken into consideration before ablating extensively and repeatedly superficial veins.

These quite disappointing outcomes in a prevalently benign disease should induce some reflections upon the need to pursue expensive innovative treatments in response to the impulses of industry, or of social media-oriented patients who seek fashionable technologies. It was estimated that varices progress with a rate of about 4% per year, with a reported 58% progression after 13 years [30]. Consequently, we could argue that the natural chronic, evolutionary, and worsening nature of this disease throughout the years nullifies the little outcome difference between expensive and inexpensive methods at short/mid-term follow-up.

In fact, encouraging new technologies in varicose vein disease should encompass cost-effectiveness analysis as a pre-requisite for their applicability and diffusion [62]. As re-treatment is an integral part of any therapy for this common disease, the vascular community could focus on mini-invasive, feasible, inexpensive, safe, and effective treatments in order to optimize efficacy and minimize harms and spending.

The estimated 60% increase of the number of varicose vein treatments, between 2013 and 2021 [63], should also impose a reflection on the appropriateness of any therapy in the public health systems, possibly choosing inexpensive methods, also in view of the expected demographic changes in the years to come.

As highlighted above, obesity is an important negative prognostic factor in CVD; recently, a multicenter prospective study [64] clearly proved, in a large cohort of 65,329 patients, that a progressive increase in body mass index (BMI) negatively affects the QOL outcomes of any varicose vein treatment at 6 months. When BMI exceeded 35, the outcomes progressively deteriorated, and above the figure of 46, treatments had extremely negative outcomes.

In addition to the data above, we observe that obese patients are usually intentionally excluded from most clinical trials regarding treatments of varices, hence the real magnitude of the negative effect of this condition on the outcomes is little known; overall, reducing a patient’s weight prior to any varicose vein procedure (as in any CVD interventional procedure) seems a logical strategy.

Furthermore, patient reported outcomes (PRO) seem to be very similar at mid-term, regardless of the treatment [48,62]. Combining technical success with PRO in the evaluation of varicose vein treatments, the differences among innovative procedures and older (stripping) or simpler and cheaper ones (UGFS) seem to approximate [48,62].

In view of the above evidence, cost/effectiveness ratio should drive the therapeutic choice in varicose disease, beyond the physician’s and patient’s preference, which actually represent the leading decisional factors [62]. With varicose vein treatment being one of the most represented active interventions in phlebology, it would be important to define the proper outcome assessments beyond the inconclusive saphenous occlusion rate. Indirectness of most literature on this subject include surrogate markers (e.g., occlusion/recanalization rate at duplex ultrasound), lack of comparison to other treatments, suboptimal reference to PRO, such as function, symptoms, and QOL [62].

Safety of treatments for lower limb varices is another relevant parameter. Interestingly, O’Donnell et al. investigated the thrombotic adverse events in 131,887 subjects who underwent varicose vein treatments. DVT incidence was as follows: radiofrequency ablation 4.4%, laser ablation 3.1%, surgery 2.4%, and sclerotherapy 0.8%. PE incidence was 0.3% in thermal ablation or surgical procedures, and 0.2% in the sclerotherapy group [65]. Similarly, neural complications after these therapies may occur, especially with endovenous thermal ablation or surgery [66]. Conversely, neural lesions in sclerotherapy incidence are reported as 0.02% [67].

Cost-effectiveness analysis may use improvements in QALY (quality adjusted life years) as a common measure throughout different health systems worldwide. QALY-based analysis of therapy for varicose veins [54,68] have gained popularity. Patient’s perspective should have a place as well, as notoriously, both technical success and failure of varicose vein treatment do not necessary correlate with PRO.

Patients’ expectations may include a durable clinical (functional and cosmetic) result, with symptom and QOL improvement, positive influence on social and working activities, low risk of recurrence, and lastly, minimal personal costs [62]. Treating physicians’ perspectives are mostly different, focusing on the success (efficacy and safety) rate. Payers’ perspectives focus on the spending issue, together with the health improvement. With an increasing number of patients wandering from one treating center to another, after undergoing some 3–4 interventions for their varices, the issue of therapy appropriateness for an extremely common, recurrent, and socio-economically relevant disease exists.

Other presumptively relevant parameters have been proposed to justify innovative and costly technologies, though they appear of limited interest within a lifelong disease assessment. As an example, tumescentless techniques cite the absence of the discomfort induced by tumescence injections (usually 4–5 in the thigh) to justify the preference towards a significantly more expensive technology. Similarly, several studies report about the difference of post-operative pain as a core issue to prefer one method over another.

Overall, a reflection on the cost issue has been recommended in recent papers [68,69]. Regrettably, current practice in varicose vein therapy is subject to several non-medical issues. Ricci, Mendoza and De Maeseneer respectively, remark with emphasis that: “new gadgets will be continuously invented, leaving unchanged the GSV closure rate”, “requirements for the introduction of a new device are far less stringent than for the introduction of a new drug”, and “in lots of countries, the health professionals’ income depends on their performance. The higher the income for a procedure, the higher the personal financial benefit… the more the cost, the better the treatment, the better the income” [70].

### 2.2. Perforating Veins

The evidence for treating perforating veins is extremely weak and several interplaying morphologic, hemodynamic, pump-related factors interact in determining the real pathogenicity of perforators. Although direct therapy of leg perforators has some evidence in advanced cases (i.e., C5 or C6 of CEAP classification), the same was considered inappropriate in the vast majority of the cases, such as in C2 or C3 cases [71]. Regardless of the complex assessment of perforator incompetence and their real pathologic role in skin dystrophies, a few variables such as the co-existence of primary or secondary varices, post-thrombotic syndrome (PTS), musculo-vascular pump dysfunction, and obesity, significantly impact the treatment outcome.

In fact, most experts propose that any perforator therapy should be deferred until the main venous causes of CVD (e.g., saphenous refluxes, deep venous hypertension) have been addressed.

Lastly, in active or healed ulcer limbs, thermal, surgical, and sclerosant treatments achieve similar occlusion and especially overlapping recanalization/recurrence rates [72]; consequently, in view of its inexpensiveness and safety, especially in dystrophic skin areas, UGFS should be preferred over all the other costly and more invasive ones.

### 2.3. Telangiectasias and Reticular Varices

Treatment of minor varicosities (telangiectasias and reticular varices, C1 in CEAP classification) has been based on sclerotherapy for decades with acceptable results, notwithstanding the very limited knowledge about the pathophysiology of these vein disorders [73]. Efficacy of sclerotherapy in minor varicosities is usually accompanied with a low incidence of side effects, thus coping with the main aesthetic concern of these specific patients [74,75]. This extremely inexpensive technique has been nowadays challenged by a few technology-based interventions (laser, cryo-laser, and cryo-sclerotherapy (CLACS), just to name a few) which require an expensive piece of equipment (laser, cooling system, and augmented reality imaging), a higher degree of setting complexity, and which probably achieve similar clearance rates [73]. The limited evidence from the few comparative studies highlight similar or better efficacy and safety for sclerotherapy in telangiectasias; furthermore, laser and other physical methods achieve significantly lower outcomes in reticular varices [73,76].

The few available consensus documents report that transdermal laser should be reserved only for patients with contraindications to sclerotherapy, with needle-phobia, or with some side effects from previous sclerotherapy [35,77]. The only PubMed-based publication on CLACS [78] reports 86% satisfactory results, which is in the range of the published outcomes of sclerotherapy [74]. As a result, the extremely higher costs and more complex setting of CLACS, in comparison to sclerotherapy, does and will represent a relevant issue in the future.

Nowadays, in cosmetic medicine, the patients’ quest for innovative, painless, and problem-solving technologies is extremely challenging. Regrettably, this demand is associated with a high patients’ aesthetic expectation and sometimes with a certain degree of psychopathology. Consequently, industries, biomedical science, and physicians are under strong pressure that often leads to expensive therapies which meet patients’ demands and not the necessary cost-effectiveness. In view of the significantly high-cost disparity and of the outcome similarity of the treatment of reticular varices and telangiectasias, the choice between sclerotherapy on one side, and transcutaneous laser, CLACS, or other physical instruments on the other side, is probably destined to be based on non-medical reasons.

### 2.4. Deep Vein Thrombosis and Post-Thrombotic Syndrome

Endovascular/open surgery interventions in deep vein diseases (DVT, PTS) still lack long-term validation studies, as multiple factors (leg/diaphragmatic pump dysfunction and obesity primarily) seem to interfere with the objective durable benefits. Similarly, the lack of a common validated hemodynamic criterion to assess iliac vein obstruction, together with the suboptimal imaging of the morphologic changes in abdomino-pelvic veins, represent further limitations prior to any decisional process in these patients [44]. Scientific data show that primary obstructive syndromes based on compression (e.g., May-Thurner) may suffer from an over-diagnosis and over-treatment issue. The majority of these syndromes, in fact, may vary in terms of morpho-hemodynamic findings dependent on different postural changes and weight conditions, both in normal and in diseased veins [79]. This variability raises the question of a more comprehensive approach to these iliac compression syndromes. Regrettably, their (endovascular or surgical) treatment is aggravated by costs and operational complexities, as well as a conclusive evidence on their long-term benefit is still under scrutiny.

Any direct intervention aimed at patency restoration in the immediate/short-term of an acute ilio-femoral DVT implies the use of multiple modalities and technologies: pharmaco-mechanical thrombolysis (PMT) is the most referenced therapeutic approach, whereas ultrasound-based procedures or alternative surgical approaches represent further therapeutic options. The evidence about the superiority of PMT in acute DVT over conservative methods is extremely limited [80].

The CAVENT study showed a good vein patency rate 5 years after PMT for iliac DVT, with some reduction in PTS occurrence, but QOL was not superior to the control group (conservative treatment) [81]. Conversely, the large multicenter ATTRACT trial showed no reduction of PTS incidence (at 2 years) and a higher complication rate when the same treatment preceded standard anticoagulation for acute DVT. Furthermore, PMT did not improve QOL nor recurrent VTE, but it improved leg pain and swelling at immediate term and reduced the overall severity of PTS [82].

After the ATTRACT trial, experts clearly highlighted how the 24-month examination revealed a generally incremental gain in the cost-effectiveness ratio for PMT of USD 222,041/QALY, which determined an overall inferiority of this treatment vs. standard anticoagulation [83].

In these cases, the long-term benefit, as to PTS occurrence, LVU incidence, etc., compared to the anticoagulation + compression option, is not only debatable, but the high difference in terms of costs should bring to a weighed reflection. Lastly, PMT in acute ilio-femoral DVT may have a relevant incidence of bleeding, which was nearly 8% (major and clinically relevant cases) in the CAVENT study [84].

The previously fashionable use of vena cava filters in acute DVT has been challenged by the significant complication rate [85,86]. A recent systematic review with meta-analysis has confirmed that filters may reduce the risk of subsequent pulmonary embolism, while increasing the risk for DVT; overall, mortality was found unaffected by retrievable cava filters. As a consequence, the indication to this procedure is being progressively restricted to specifically selected cases [87].

In 2011, an analysis of VTE prevalence in the USA showed a 33% increase in the 5 years to come, and the authors predicted an alarming figure of 1.8 million patients affected by VTE by 2050 [88]. These data confirm once more the need for a major appropriateness in the selection of venous procedures in VTE, reserving higher complexity/cost therapies to specific cases.

In fact, a reappraisal of invasive treatments in PTS has been recently highlighted [89]. In view of the contrasting evidence for venoplasty ± stenting in PTS patients [90,91,92], this procedure is being reserved to selected cases where other treatments have failed and the main risk factors (e.g., obesity, pump dysfunction, thrombophilia) have been already addressed, with little benefit.

More specifically, the decisional process concerning endovascular/surgical intervention in PTS limb should take into account a series of pertinent factors, which are reported in the following Table 5.

Together with the ascertained role of compression in PTS [93], interestingly, inexpensive low-dose acetylsalicylic acid (ASA) showed to significantly decrease, by more than 30%, the risk of recurrent thromboembolism (and of cardiovascular events), without increasing the risk of bleeding [94]. Furthermore, a recent systematic review and meta-analysis surprisingly documented that ASA was non-inferior to anticoagulants in VTE prophylaxis in hip and knee surgery [95].

Venoplasty and stenting in deep vein (iliac mainly) chronic obstruction shows a significant difference in terms of technical success for secondary-assisted patency vs. primary success rate [35]. This need for re-intervention in a quota of patients to maintain the mid/long-term outcomes entails additional costs which reduce the cost-benefit value of endovenous procedures in PTS.

In fact, experts have indicated that endovenous iliac recanalization with stent placement is appropriate solely in severely affected PTS patients who experience failure of proper conservative management [96]. This is a reminder here that an accurate endogenous procedure in the ilio-caval segments ideally requires the use of intravascular ultrasound for a more detailed anatomic assessment [96]. This additional expensive technology surely adds some relevant information but also, it entails a higher expense.

Surgical procedures for deep vein isolated reflux of primary or post-thrombotic nature have been recommended in extremely selected cases, due to the debated cost-effectiveness of this complex care. In fact, these procedures require a specific expertise and the overall available evidence regarding their outcome strictly depends on the type of intervention, and it is still limited [35].

Of interest, at 6 months follow-up, patients with PTS may significantly benefit from a surveilled educational approach, in terms of QOL and symptom severity, and the reported improvement was higher when an exercise training was added [97]. More generally, a few patient-centered conservative and inexpensive interventions have shown to improve the course of CVD: low-calorie nutrition, adapted physical activity, lifestyle changes and self-care, physiotherapy (compression stockings above all) and rehabilitation, and psycho-social support [98]. As the authors pointed out in their review, the limited interest in pursuing adequate studies on rehabilitation and lifestyle measures determines their yet suboptimal evidence. Caregivers’ lack of information about these conservative interventions may represent an issue to overcome in the future.

Compression stockings have always had a quite high level of evidence, both in acute DVT therapy and as chronic treatment to reduce subsequent PTS and CVI signs and symptoms at mid- to long-term. A critical revision of compression’s role in DVT and PTS patients has recently spread within the scientific community; in fact, due to the negative outcomes of the SOX trial [99], a major reappraisal of compression’s role has been suggested in these cases.

This well-referenced study significantly contributed to downgrade the evidence for compression stockings in PTS prevention, after an acute DVT episode. In agreement with several authors’ reflection [44,100], we speculate here that the following biases may have influenced the final results of the SOX trial: (a) the non-immediate, but delayed (within 14 days) adoption of the compression stocking in the treatment arm, (b) the misleading use of a placebo stocking, which anyway has a proven psycho-physical effect on the limb, (c) the suboptimal assessment of the patient’s compliance to the routine use of the stocking, which was delivered by mail, without any direct counselling from the caregivers, (d) the different anticoagulants used throughout the two groups, and (e) the required minimal duration of compression regime (3 days a week), which is not coherent with all the pertinent literature.

As a result, a more coherent discussion and more literature data are expected, so as to corroborate or disprove the role of compression stockings in DVT and PTS.

### 2.5. Anticoagulants

Direct oral anticoagulants (DOAC) employment has been encouraged in several venous diseases, though uncertainties may exist as to a few indications.

A recent systematic review and meta-analysis [101] on 4509 patients with cancer-associated DVT (1868 individuals receiving DOACs and 2641 receiving low molecular weight heparins (LMWH)) found a major bleeding risk with DOAC (relative risk 1.78, 95% confidence interval 1.11–2.87). Overall, VTE risk was lower for DOAC, hence the latter may have an indication in cancer-related DVT patients, provided they have a low risk of bleeding.

When expanding DOAC use in SVT or in thromboprophylaxis, a careful balance between benefit and possible adverse events should be considered. As an example, a recent systematic review and meta-analysis [102] on 116,289 patients found no relevant differences between DOAC and dicoumarols in terms of gastrointestinal bleeding. Furthermore, the absence of specific reversal agents for DOAC should represent a caution criterion as well. Therapy with DOAC in SVT represents a controversial indication [103], and probably further studies should corroborate this approach, in opposition to the validated protocol with LMWH and compression. Stratification of SVT patients as to risk factors would probably benefit treatment selection, preferring DOAC only in higher VTE risk patients [44,104]. Overall, in the last decades, an extremely large number of studies have addressed DOAC and LMWH employment, mostly thanks to the impulse coming from the industries, with new pharmaceutic products overriding one over another. Some contrasting evidence emerges from all these studies, hence the safety issue and the need for more tailored and cost-effective anticoagulation regimes are emerging.

### 2.6. Pelvic Congestion Syndrome

Pelvic Varicocele, with related symptoms and signs (pelvic congestion syndrome, PCS), still lacks a unanimous definition and clinical/instrumental diagnostics. Trans-catheter sclero-embolization is the therapy of choice, though the published evidence has been considered quite low [105].

Due to the complexities, costs, risk of complications, and due to the uncertain modalities for the assessment of the outcomes of any interventional treatment, better trials are warranted [105]. Future studies on sclero-embolization should focus on the agreed modality to perform both the treatment (coils and/or sclerotherapy) and the outcome assessment, beyond the classic patient-reported changes in pelvic pain. Longer-term results in homogenous populations and comparative studies with conservative treatments would be desirable as well. More importantly, the definition of proper indications to this radiologic procedure should be addressed; in fact, an invasive treatment could be taken into consideration when relevant chronic pelvic pain (and dyspareunia), possibly with related varices in the lower limbs, is accompanied with pelvic venous hypertension [105,106,107].

Management of PCS may be complicated by the possible concomitance of pelvic vein compression syndromes (e.g., May-Thurner or nutcracker): these are not infrequent in patients with PCS and may reduce the therapeutic value of the classical gonadal vein sclero-embolization

### 2.7. Leg Venous Ulcer

Leg venous ulcer (LVU) represents a relevant socio-economic burden worldwide, with an average of 1–2% expenditure of national health systems [63]. In the USA, LVU annual cost was estimated to be about $15 billion USD [108]. Leg venous ulcer in most cases derives from varices, PTS, and from several influenceable and detrimental co-factors, such as obesity, aging, traumas, disabilities, musculo-vascular pump dysfunctions, lymphatic drainage impairment (e.g., after extensive great saphenous stripping or repeated skin infections), sedentarism, inflammatory processes, diabetes, and nutritional state. A major attention should be given to the prevention of LVU, mostly through an adequate control of these causal diseases and of the concomitant deleterious conditions, as above.

A general lack of agreement among various guidelines concerning the clinical practice in LVU management has been shown [109], which is mostly due to the heterogeneity of studies and literature conclusions. Amidst this suboptimal evidence, compression still emerges in its different forms, as the therapy that proves to heal about 80% of LVU within 6 months [110]; similarly, early treatment of lower limb varices proved to add a significant benefit as to ulcer healing and recurrence time [111]. Regardless of this data, the large majority of LVU is usually treated with other methods by various kinds of caregivers worldwide, very often without an adequate compression regime, or any reference to varicose vein therapy.

In fact, patients under compression have a better healing rate/speed, in comparison to those without compression [112]. Treatment of LVU without compression was reported to have a healing rate of 20–26% at 12 weeks, which rose to 36–42% in a dedicated leg ulcer setting [113]. Also, LVU recurrence rate is higher, up to 70%, when compression is not practiced after ulcer healing [110].

Healing costs of LVU were estimated to be about 2065 USD and 4330 USD respectively, when compression was used or not included [113]. Many of the caregivers (nurses, physiotherapists, or physicians) may have a limited practice of compression therapy, equally performing no treatments for varices, as well as they may have a prevalent ulcer care culture based on topical dressings and “advanced” medications. The resulting expensive, and possibly less effective, treatment of LVU by this type of personnel may aggravate the overall social and economic costs of this disease, which has a prevalence of up to 4.3% in the general population [114] and of 5% in the elderly [115], with an estimated 50% recurrence rate at 5 years [63].

In general, wound management is addressed by healthcare providers/payers with some neglect; hence, a higher quality in the cost-effectiveness management of LVU should be researched, also in view of its socio-economic impact [116]. The highest cost in LVU community-based therapy is probably represented by the treating nurse, which was shown to drop from 16,371 to 3239 USD when caregivers receive an adequate training program [117]. This and other issues have raised the awareness towards the need of a major patient’s involvement in self-management of ulcer care, possibly through adjustable compression wraps [118]. In fact, this medical device proved to be cost-effective in LVU in a recent randomized controlled trial [119], as well as their role in LYM has been established in past studies [120].

The cost/effectiveness of advanced wound dressings in LVU is still debatable and their use is not recommended as a general routine [121,122,123]; moreover, a Cochrane review has highlighted no relevant benefit when complementing advanced dressings to compression in LVU care [124]. A possible higher efficacy of advanced dressings over conventional older ones was reported in larger ulcers and on patients’ symptoms [125].

In fact, advanced wound care matrices may add relevant costs to LVU management [126], when compared to sole compression treatment. Conversely, an Australian study showed a cost saving of about $1.4 billion AUD after 5 years when LVU-affected patients were provided with compression therapy products [127].

In terms of expenditure saving, a recent randomized controlled trial showed that an adjunctive 12-week supervised exercise training over compression alone provided both a significantly more rapid LVU healing and a net benefit of about 1485 GBP per patient at 12 months [128]. In a LVU-dedicated service, conservative treatment with complementary surgical treatment in nearly 50% of the cases achieved a 60% rate at a mean time of 122 days, whereas 20% had healing with recurrence in about one year’s time and 20% did not heal during the same period [129]. The comprehensive cost of the treatment was respectively 10,563 USD and 33,907 USD for healed and non-healed ulcer patients. Outpatient facilities and visiting nurses represented the highest costs, but when inpatient management was necessary (mostly for ulcer infection), the cost per patient was 33,629 USD (*p* < 0.001). Surgery in general had a 35% increase of costs in comparison to conservative treatment (*p* < 0.06), but specifically, deep vein surgery significantly raised the costs over medical treatment or superficial surgery. Furthermore, when skin grafting was performed in combination with deep vein intervention, the patients had a five-fold increase in costs compared with conservative or superficial surgery groups. Of concern, complications related to deep vein outflow surgery (33% of the treated cases) raised the costs to 71,526 USD per patient.

These data emphasize the need for a very limited indication to major surgical procedures in LVU patients, as well as for a strict control of infections. In 2016, a detailed review on the clinical evidence for treatments in LVU [130] examined healing rates, recurrence rates, quality of life, and adverse effects. Overall, compression treatment, superficial venous surgery, and pentoxyfilline achieved the best outcomes, while vacuum technology, skin grafting, and laser had a “very low” level of evidence. As for LVU recurrence, medical compression elastic stockings were clearly shown to be beneficial in the short/mid-term [131]. This data reinforces the need for an adequate use of this medical device in these patients, even more when combined with a series of lifestyle modifications and self-care measures [132].

Regrettably, the level of evidence for compression in LVU recurrence varied from 2B to 1A throughout several international guidelines, which may reflect a series of biasing factors when dealing with compression in general [44]. In fact, beyond the type device used (e.g., stocking, bandage, adjustable compression wraps, intermittent pneumatic compression) and beyond the operator’s skill, the effects of compression strictly depend upon the pressure that is exerted on the treated limb, and only secondarily on the stiffness of the used material. These variables are seldom measured and/or reported in the published clinical trials, which undoubtedly jeopardizes the value of their outcomes.

Microcirculatory stasis is at the basis of any LVU, yet scientific research mostly focuses on the relevance of venous reflux and/or residual obstruction as the only causal factors. In fact, stasis may derive from the above-mentioned morpho-hemodynamic findings, which are typical of varicose veins and PTS; equally, in the presence of normal veins, microcirculatory stasis and tissue alterations may originate from dysfunctional calf/respiratory pumps, obesity, drugs such as calcium channel blockers, alfa-lytics, and other factors.

Unfortunately, the near totality of the studies on LVU management lacks a detailed analysis of those associated clinical conditions which definitely represent relevant prognostic factors. When leg pump dysfunction was specifically examined, it was found to be an extremely detrimental factor in ulcer healing time [133].

Together with the diagnostic/therapeutic procedures aimed at reducing ambulatory venous hypertension, the control of all the other possible stasis-generating factors would be desirable to improve LVU healing rate and especially recurrence rate. Lastly, the use of expensive therapies such as advanced topical dressings or deep vein procedures should be reserved to selected, non-responding cases.

### 2.8. Lymphedema

Lymphology is erroneously considered a sort of Cinderella discipline within vascular medicine, and hence it is probably in need of a reappraisal of the current knowledge and even more in search of further diagnostic and especially therapeutic options. In fact, LYM diagnosis and treatment are still based on many “old” concepts, as well as the outcomes of whichever therapy is still of limited value in the mid- to long-term [134]. Lymphedema shares with CVD many causal and aggravating factors, such as obesity, calf pump and breathing dysfunctions, edema-generating drugs, and erroneous nutritional/lifestyle factors.

Beyond the unanimously practiced complex decongestive treatment (CDT), a comprehensive treatment of LYM could also address these epigenetic pathways within the so-called translational medicine, hence through a multidisciplinary approach [39]. As obesity dramatically influences CVD, equally, this pandemic metabolic degeneration alone suffices to impair lymphatic function in normal subjects. Obesity does represent a strikingly important risk factor to generate or worsen LYM, thus nullifying many of the therapeutic efforts [39,42,135].

Surprisingly, little attention is usually given to weight control in the vast majority of the publications regarding LYM management, though a series of recent narrative reviews highlighted how obesity mechanically and metabolically impacts lymph flow, lymph formation, fibroadiposis formation, and more generally, LYM onset and course. Similarly, a series of publications have remarked the importance of nutrition, nutraceuticals, microbiota, and lifestyle as factors which may positively or negatively impact lymph stasis [39,42,136].

Lymphedema is an inflammatory CDD, where CLGCI plays a fundamental role in the derangement of lymphatic drainage and in edema/tissue deterioration. Several biochemical alterations, such as macromolecules accumulation and consequent inflammation, hypoxia, oxidative stress, and fibroadiposis, contribute to LYM pathophysiology.

Literature about LYM shows inhomogeneity and discrepancy among the few available guidelines and recommendations, which makes them inadequate to provide sound bases for an agreed clinical practice [137,138].

Whether the few current innovations will give relevant beneficial outcomes in LYM management is still to be established; however, the recent international consensus document clearly admits that “no treatment method has really undergone a satisfactory meta-analysis…and some degree of uncertainty, ambiguity, and flexibility along with dissatisfaction with current LYM evaluation and management is appropriate and to be expected” [134]. Keeping in mind that any chronic limb edema features a lymphatic system organic/functional derangement and considering that the lymphatic system does play a role in several CDD, a major effort to improve lymphology science would benefit the vascular and non-vascular community [39].

Primary LYM objectively depends on genetic predisposal, but epigenetics (nutrition and lifestyle above all) were shown to influence both onset and evolution of this disease. Equally, secondary LYM seems to be not solely a mere consequence of a mechanical blockage (lymphadenectomy). In fact, a genetic susceptibility was found for women with connexin47 genetic mutation, who express a higher risk of breast cancer-related LYM [139].

Overall, management of lymphatic insufficiency could benefit from translational and integrative medicine with an in-depth research on the core inflammatory process of lymphatics, nodes, and especially of tissues. In fact, in the last decades, very few innovative and relevant therapeutic proposals have been introduced in LYM management. In fact, therapy of lymph stasis is still based on a compound of multiple modalities which are basically finalized to favor fluid centripetal squeezing, with little to no chance of addressing the core lymphatic, lymph node, and tissue morpho-functional disease. Compression treatment is considered the cornerstone of this palliative approach (CDT). This simple therapeutic modality was proven decades ago and still proves to be an effective treatment in LYM [36,117,134], which frustratingly testifies the need for improving the scientific research on this subject, possibly targeting lymphangiogenesis, anti-inflammatory pathways, and the relative epigenetic factors, as described above. International consensus documents on LYM report about interesting outcomes deriving from microsurgery as well, which aims at addressing the basic cause of lymph stasis [36,134].

Of interest, the strict relationship between the lymphatic system and a series of CDD (namely neurodegenerative diseases, atherosclerosis, cancer, autoimmune diseases, diabesity, infective diseases) may represent an opportunity for vascular specialists to contribute, through translational medicine, to the progress of scientific research in these remarkably important areas [39,140].

Self-management in LYM patients has become a relevant issue in terms of cost-effectiveness, and it represents a possible opportunity to improve outcomes and sustainability of care provision in this chronic, life-lasting, and disabling pathologic condition [141]. Compression by adjustable compression wraps is being assessed and validated in LYM [117,142] and in LVU [116], proving to be an interesting option, both in terms of economics/ergonomics and as to the achievable clinical outcomes. Similarly, lymphedematous patients’ awareness about the negative and positive influence of lifestyle and nutritional factors could play a fundamental role to optimize LYM management [39,42,143].

With LYM being secondary most often related to cancer treatment, further complexities and spending issues may affect the management of these patients. Incidentally, it was found that after breast cancer therapy, patients with LYM have higher medical costs in comparison to those without LYM (USD $23,167 vs. $14,877, respectively) [144]. Changes in health policy regarding prevention and early detection of LYM in these specific patients may have a positive clinical impact and worthy ergonomic/economic repercussions [145].

The role of surgery in LYM, both intended as tissue excision or as a microsurgery procedure, is debated. Regardless of the relevant amount of literature on this therapeutic option, most reviews highlight a great lack of homogenous data [44]. A few contrasting reviews claim quite favorable outcomes [146], or, oppositely, negative results [147], for derivative or excisional surgery in limb LYM. Of particular interest is the fact that the majority of the studies on surgical therapy in LYM invariably report about the need for an associated intensive pre-post-operative conservative treatment, which may ultimately bias the outcomes.

Derivative or reconstructive microsurgery, under the form of lymph node transfer, or lympho-venous anastomoses, has obvious advantages (and better outcomes) over reductional/excision surgery, as the latter frequently requires life-long conservative therapy. However, better results have been generally reported when surgery was applied in earlier stages of LYM, where conservative therapy equally achieves its best outcomes. In absence of sound evidence, most guidelines orientate the best practice, suggesting surgery in those cases which do not respond to cycles of conventional CDT [36,134].

Economics of lymphatic microsurgery in breast cancer-related LYM were examined in a specific paper [148]. Notwithstanding a few possible criticisms concerning the authors’ calculations of conservative treatment (probably overestimated) costs, overall microsurgery proved cost-effective when it was followed by discontinuation of ongoing conservative therapy, which is unlikely to occur in the vast majority of these patients who are affected by a life-long disease.

Again, the relevant costs of surgical interventions, the limited evidence, combined with the low availability of centers where this treatment is adequately performed, question the real appropriateness and feasibility of surgery in the vast majority of lymphedematous patients.

Overall, the appropriateness of the criteria used in the provisioning of healthcare in phlebolymphology has been recently addressed in a consensus document [71]. Segmental saphenous refluxes, incoherent symptoms, and edema were examples of debatable or incorrect indications to saphenous venous ablation, or to stenting for ilio-caval segments. Similarly, an incidental finding of ilio-caval compression at imaging was not considered an indication to stent placement. Symptomatic C4–C6 patients were classified as appropriate candidates for iliac vein stenting, but only when in the presence of an obstruction greater than 50%. However, likely, in C4 (and probably in C5 as well), more evidence is needed. Furthermore, stenosis degree is not a fully validated parameter and it is a debated morpho-hemodynamic finding, due to the frequent biases induced by biplanar imaging (computed tomography and magnetic resonance) and posture-induced variations in the iliac segment [79]. Surprisingly, the authors had to overemphasize that healthy non-refluxing saphenous veins are not appropriate for treatment, which reinforces the need for a reappraisal of the appropriateness of venous diagnostic/therapeutic pathways in reimbursement-based healthcare systems.

When debating about appropriateness, the safety of medical interventions represents a main topic to take into consideration. The literature shows that medical errors together with diagnostics/treatments may be considered the third leading cause of death in the USA [149]. Similarly, in a recent review, 20.6% of medical care was rated as unnecessary [150]. This “overtreatment” may pertain to a few venous diseases as well, regarding diagnostics, drugs, and therapeutic technologies [71].

Past AVF President M. Passman has recently underlined some need to reinforce ethical principles in phlebology, so as to achieve a higher degree of appropriateness in the diagnostic and therapeutic decisional process [151].

## 3. Eminence-Based and Reimbursement-Based Phlebology and Lymphology

Scientific literature about phlebolymphology is often crowded with publications by eminent authors, due to logistic, economic, and cultural tradition reasons. These groups of scientific leaders consequently have the power to establish sound bases of the consensus documents and best practice. Regardless of the correctness of the scientific production, the concentration of this scientific “power” in a limited number of centers may create the risk of what has been called “eminence-based medicine” [152].

Phlebolymphology diagnostic and therapeutic pathways may significantly differ among countries or continents, as to the existing national public/private/insurance health systems. Where most medical management is based on insurance reimbursement, patients’ diagnostics and treatments are influenced by the reimbursed fees (intuitively, the higher the reimbursement, the higher the procedure practicability) and by the acceptance of technologies/drugs by the insurance companies. Conversely, a tendency towards a cost-efficacy assessment of each intervention is contemplated where the decisional process is not influenced by income issues.

Assessing economic issues in medicine includes a plethora of factors which interfere with the final judgement of the cost/efficacy ratio for each procedure. Examples of proper examination of these issues exist in phlebolymphology literature (National Institute of Clinical Excellence guidelines, Cochrane reviews, just to name a few). Recent reviews of cost-effectiveness of varicose vein treatments [68], or about dressings and topical agents for treating VLU [118], properly address the need for targeting best practice at convenient costs.

## 4. Flaws of Patients, Health Professionals, Industries, and Politicians

Generally, patients prefer pills and technology to the due changes in lifestyle, nutrition, and in psycho-social conditions. Regrettably, there is a similar tendency in vascular physicians, who tend to prescribe drugs and use technology in healthcare systems more than pursuing a tiring and energy-consuming educational process for patients with chronic venolymphatic diseases.

This unsolved issue of patients’ choice to maintain the detrimental lifestyle and nutritional habits tends to match the current management practices of physicians and healthcare professionals. In fact, patients have delegated their own health to doctors and pharmaceutical and techno-medical industries. In cases of LYM, LVU, and CVI, patients prefer drugs and medical interventions to the search of basic lifestyle healthy measures such as: weight control, low-carb and nutrient-dense food nutrition, exercising and postural rehabilitation, other practices exerting a systemic anti-inflammatory action (such as breathing, stress control, adequate sleep etc.), and other risk factors’ reduction (smoking, alcohol, etc.).

Changes would be desirable from excessively passive physicians and patients, both to improve the therapeutic outcomes and to rescue the future of healthcare in terms of costs and social organization.

Medicine in general relies on scientific research evidence, which leads to proper healthcare. Of great concern, in 2013, a reliable examination of the randomized controlled trials regarding 3000 currently applied treatments [153] highlighted the following discouraging results: 11% of the investigated therapies were categorized as beneficial, 24% were categorized as likely to be beneficial, 7% as having trade-off between benefits and harms, 5% as unlikely to be beneficial, and 3% as ineffective or harmful. The residual 50% were rated of unknown effectiveness, as they were not supported by randomized controlled trial evidence.

Overuse of medical services has been generally recognized worldwide [154] and a movement towards a wiser choice of diagnostic tests and therapies is spreading valuable suggestions [155,156]. Beyond the possible patients’ harm derived from these over-diagnostics and overtreatments [149], overuse contributes significantly to the total spending for healthcare worldwide. In the USA, about $270 billion USD were spent due to medical overuse in 2013 [154], and in a few countries, healthcare expenditure represents the greatest threat to the financial balance, which will deteriorate with the increasingly aging population.

Of importance, medical industries may significantly influence scientific research, as it was repeatedly shown several years ago [21,157]. A recent Cochrane review [24] confirmed this trend, and a sort of “publication bias” was claimed by a few authors [22].

Medicine in general is dedicating a specific attention to the problem of overuse [158], hence it is expected and desirable that in the future, phlebology and lymphology may also benefit from this cultural re-appraisal in order to reduce any low-value diagnostic and therapeutic intervention.

Scientific evidence undoubtedly has the merit to harmonize the medical approach worldwide with a high level of trust. Additionally, the epistemic complexity of EBM should be discussed in order to exploit EBM in an ethical and cost-effective way. Scientific evidence drives medical practices, which dramatically impact human lives. Since science is fallible, and can be biased and subject to controversial influences, the combination of EBM with physicians’ elaboration based on patients’ comprehensive assessment would add value to the daily medical practice.

In fact, EBM provides standard recommendations, based on the available scientific knowledge, and with the concept of standardization being inherently affected by weakness and robustness, medical practice should be “a transitive relationship between a truth of reason and a truth of fact, hence among evidence, doctor, and patient” [159]. Having this in mind, the singularity of each subject should be regarded as a relevant issue, since medicine is not an exact science. The so-called “statistical regularity” of medical evidence cannot assume that conventional truths based on certain constant findings are irrefutable, especially in light of the infinite singularities of the patients.

Undoubtedly, a physician’s task is complex in view of the many variables which interfere with the final decisional process. The combination of the scientific “temporary” truth with a pragmatic patient-centered approach may be an “epistemically acceptable” option, and the caregivers’ intellectual autonomy, science, and conscience may cooperate in this process [160].

## 5. Conclusions

The reductionism which characterizes part of current biomedical science, too often focused on drugs and technology, has undergone major criticism by several authors [37,161,162,163]. Patient’s lifestyle, nutrition, and psychosocial conditions, together with the environmental changes, are key factors in health and disease processes.

Translational medicine, epigenetics, and integrative medicine undoubtedly represent future fields of interest in the management of venous and lymphatic diseases. The inclusion of these advanced approaches in the panorama of current phlebolymphology science has the potential to be extremely beneficial and cost-effective. Unfortunately, most translational medicine research needs an average of 17 years to be put into practice [164], hence the scientific community is required to bridge this gap with some decisiveness.

A few recent publications [165,166] have rigorously assessed the evidence, from the available literature, about the treatment of patients with CVD of the lower limbs. They highlighted an extreme heterogeneity of the available studies, together with the lack of proper trials on conservative treatments related to lifestyle and nutrition. Endovenous interventions for varices of the lower limbs showed no statistically significant differences in terms of outcomes, both in comparison to surgery and among the same procedures; furthermore, they were considered “not yet rigorously tested”.

Chronic veno-lymphatic diseases are likely destined to have a growing prevalence, due to their strict correlation with age and with the increasingly frequent weight excess and disability; thus, a careful strategy about cost-effective diagnostic and therapeutic means is required, in view of the increasing longevity.

A comprehensive approach which includes the patients’ major awareness and self-management may reduce the future higher burden of chronic veno-lymphatic diseases, currently estimated as 2% of the total budget (just for CVD), on our healthcare systems [167].

The integration of current phlebology and lymphology knowledge with a wider functional and integrated approach to the entire psycho-biologic human reality may contribute to significant improvements against physicalism, reductionism, and caregivers’ and patients’ inadequate decisions. Also, a higher degree of awareness in health professionals may benefit patients and the future of medicine. An ethical support of industry is desirable, together with a much more impactful health education for patients. The final aim would ultimately be a more patient-centered approach to chronic venous and lymphatic diseases and the consequent socio-economic sustainability of their management.

The exponential increase of modern procedures, more in phlebology than in lymphology, has several causes, as we detailed above; invariably, the final effect is the extension of these technologies and drugs into alternative areas, due to inappropriateness, excessive indication liberty, or due to other unethical issues [151].

Citing M. Meissner, “without being victims of a tyranny of the evidence, but integrating it with clinical expertise, the patient’s values and preferences”, vascular specialists may reach the goal of a more balanced decisional process between risks and benefits, especially before embarking in costly new therapies [168].

After Gillon, medical practice is considered ethical when it respects a few principles, which are listed in the following Table 6 [169].

A recent systematic review of all Cochrane reviews pointed out that the quality of evidence in medicine did not change significantly in the last 17 years [170]. The authors’ conclusion indicates the need for a major reappraisal of scientific research, focusing on several biases and flaws.

In fact, it is acknowledged that a few health systems may have a higher degree of connection with industry support, due to a series of purposes, such as the scientific research advancement and educational projects. Regrettably, medical association leaders may exhibit a significant tie to industries; in a recent study, this linkage was in the range of 130 million USD between 2017 and 2019 for 235 (80%) out of the 293 investigated USA chief medical leaders [171]. This relevant relationship may somehow undermine the independence of biomedical science, “adding weight to calls for policy reform”, as the authors state.

Of interest, Cochrane reviewers also found that sponsored trials get more favorable results than non-sponsored ones, which is not explainable by standard risk biases [24]; moreover, a recent Australian cross-sectional study highlighted that 24% of guideline writers had potentially relevant undisclosed ties to drug companies [172].

While evidence keeps its central role in guiding medical diagnosis and therapies, patient-related variables and socio-economic issues should also be factored into the decision processes to determine the best medical pathway for each specific case. In clinical practice, merging evidence, ethics, and a more comprehensive view of patient’s clinical condition could result in a higher degree of appropriateness, cost-effectiveness, safety, and ultimately, efficacy.

Overall, the future clinical approach to phlebology and lymphology could include a patient-centered vision, which questions the root pathophysiology mechanisms of a certain disease; as a consequence, therapies could be based on drugs, surgery, and technologies, but always in combination with good nutrition (and nutraceuticals when needed), correct lifestyle, and proper stress management. Lastly, a strict reference to cost-effectiveness and appropriateness of the available diagnostic/therapeutic means should be an integral part of any medical decisional process.

## Figures and Tables

**Table 1:** Main biochemical processes of chronic low-grade cellular inflammation and aging.

Production of free radicals in excess (oxidative stress), with injury of cell components, primarily mitochondria DNA. Consequent altered mitophagy and cell apoptosis, reduced mitochondrial biogenesis and functionality. Excess of advanced glycation end-products (excess of glycation processes). Alterations in chromosomes (degeneration of nuclear DNA, friction, shortening of telomeres, replication errors, etc.). Alterations in micro-RNA production. Proteasome degeneration (accumulation of malfolded/altered intracellular proteins).

**Table 2:** Integrative medicine interventions.

Caloric restriction/quality nutrition (reduced intake of refined carbohydrates and hydrogenated and processed fats, above all)/intermittent fasting. Adequate physical activity. Intake of nutraceuticals (including compounds, such as polyphenols and senolytics) with beneficial metabolic-epigenetic features. Regulation of the psychoneuroendocrineimmunology (PNEI) system by means of various procedures, such as parasympathetic stimulation, breathing, meditation/mindfulness, sleep regulation, and psychotherapy. Microbiota/microbiome regulation through targeted probiotics and nutrition, favoring re-balance of the microbiological flora (mainly in the gut). Activation of hormesis pathways, which favor beneficial biochemical processes thanks to short-term/low-dose exposure to biophysical/biochemical stimuli, which would be otherwise toxic or lethal at higher doses. So-called “mitochondrial” therapy to improve biogenesis and proper function of mitochondria, together with an improved mitophagy.

**Table 3:** The most common cognitive biases.

Confirmation bias: the greater weight given to any information that confirms our orientation, neglecting contrasting data. Overestimation of own skill and technical possibilities and underestimation of the possible confounding factors. “Bandwagon” effect: an impulse to follow common, previously popularized knowledge, instead of developing a critical sense. Dunnig–Kruger effect: denigration of other contrasting, presumed inadequate, science providers. Anchoring effect: the human mind’s trend to overestimate the first information it possesses, even in contrast with subsequent information. Fallacy of the probability calculation by which researchers expect a due result.

**Table 4:** Epigenetic factors which may deteriorate chronic venous and lymphatic diseases.

Obesity/overweight High carbohydrates intake (high circulating insulin) Low intake of natural anti-inflammatory compounds (polyphenols, omega 3 polyunsaturated fatty acids, vitamin C, etc.) Sedentarism Decreased and disrupted sleep Hyperactivation of stress axes Excessive alcohol consumption Prolonged standing or sitting Reduced/altered breathing Intake of drugs (e.g., calcium channel blockers, alpha-lytics, corticoids) which negatively affect venolymphatic function Environment pollutants (e.g., endocrine disruptors)

**Table 5:** Decisional factors in endovascular/surgical intervention for post-thrombotic syndrome.

Concomitant coexistence of other stasis-favoring conditions (e.g., pump dysfunction, obesity) Procedure-related costs and technical complexities Availability of trained specialists Instrumental morpho-hemodynamic diagnostic findings Concomitant collateral pathways’ functionality as natural bypass Coexistence of relevant thrombophilia Achieved/achievable outcomes with proper conservative treatment

**Table 6:** Principles for an ethical medical practice (after Gillon [169]).

Patient’s autonomy of decisions regarding healthcare procedures Justice (burdens and benefits of new treatments equally distributed in our society) Beneficence (the intent of doing good for the patient) Non-maleficence (the diagnostic/therapeutic procedure has a risk/benefit ratio which does not contemplate any pre-determined harm to the patient

## Appendix A

**Headings and Keywords Used in the Literature Search**

**Headings:** evidence-based medicine, translational medicine, integrative medicine, cost-effectiveness, pharmacoeconomics, overuse, cognitive bias, patient-reported outcomes, quality of life, quality-adjusted life years, phlebology, lymphology, aging.
**Keywords:** chronic venous disease, deep vein thrombosis, superficial vein thrombosis, venous thromboembolism, venous ulcer, varicose veins, post-thrombotic syndrome, pelvic congestion syndrome, lymphedema, telangiectasias, duplex ultrasound, anticoagulation, endovenous treatment, pharmaco-mechanical thrombolysis, venoplasty, venous stenting, thrombectomy, laser, stripping, MOCA, glue, sclerotherapy, foam sclerotherapy, phlebectomy, compression therapy, bandaging, stockings, wound dressing, complex decongestive therapy, lymphedema surgery, rehabilitation, lifestyle, nutrition, stress, obesity.
